# What Will Be the Result

**Published:** 1872-05

**Authors:** 


### What will be the Result

If the Cumming's patent is not sustained by the United States Court? Will the use of rubber for artificial dentures largely increase after the expiration of the Goodyear patent? Or will it gradually decrease, as it has been doing for the past three years?

We hope and are inclined to think the latter. A great many people will pursue a given course in the face of opposition and embarrassment, that they would not desire to do under other circumstances at all. And we have many and unmistakable evidences that there is a growing opposition to the use of rubber, especially with the profession of Ohio. Persons who in former years used gold, platinum and silver, are returning to them, and those who have never used them are anxious to learn. Many are learning that immense mischief has been, and is being done by rubber, yet some very intelligent members of the profession, in other matters, utterly refuse to recognize any thing of the kind, and claim that it is entirely improbable that any injury could be effected by it. How such assertions can be made in the very teeth of the facts that are presented to the observing practitioner almost every day is not very clear. The mucous membrane in nine cases in ten will present more or less irritation—clear and unmistakable, such a condition as is not found to exist with any other material used in the construction of artificial teeth.

